# ‘The doctors were more knowledgeable about what I had’: patient views on the value of point-of-care tests for managing respiratory infections. A qualitative study in European primary care

**DOI:** 10.3399/BJGPO.2024.0139

**Published:** 2025-06-04

**Authors:** Marta Wanat, Melanie Eugenie Hoste, Marilena Anastasaki, Femke Böhmer, Annelies Colliers, Maria Gkamaletsou, Christin Löffler, Christos Lionis, Lile Malania, Mala Shah, Anja Wollny, Akke Vellinga, Christopher C Butler, Alike W van der Velden, Sibyl Anthierens, Sarah Tonkin-Crine

**Affiliations:** 1 Nuffield Department of Primary Care Health Sciences, University of Oxford, Oxford, UK; 2 Department of Family Medicine and Population Health, University of Antwerp, Antwerp, Belgium; 3 Laboratory of Medical Microbiology, Vaccine & Infectious Disease Institute, University of Antwerp, Antwerp, Belgium; 4 Clinic of Social and Family Medicine, School of Medicine, University of Crete, Crete, Greece; 5 Institute of General Practice, Rostock University Medical Center, Rostock, Germany; 6 National Center for Disease Control and Public Health, Tbilisi, Georgia; 7 Amer Science Management LLC, Tbilisi, Georgia; 8 Pharmaceutical Care Research Group, School of Pharmacy, University College Cork, Cork, Republic of Ireland; 9 School of Public Health, Physiotherapy & Sports Science, University College Dublin, Dublin, Republic of Ireland; 10 Julius Center for Health Sciences and Primary Care, University Medical Center Utrecht, Utrecht, The Netherlands; 11 Health Protection Research Unit in Healthcare Associate Infections and Antimicrobial Resistance, University of Oxford, Oxford, UK

**Keywords:** point-of-care tests, general practice, qualitative

## Abstract

**Background:**

Point-of-care tests (POCT) can support diagnosis of patients with community-acquired acute respiratory tract infections (CA-RTI) in primary care and thereby reduce uncertainty whether antibiotics may benefit patients. However, successful roll out of POCTs need to be built on a deep understanding of patients’ perspectives on the place of POCTs in patient-centred care.

**Aim:**

To explore patients’ perceptions of the value of POCTs during consultations for CA-RTI.

**Design & setting:**

A qualitative study using semi-structured interviews in Belgium, Republic of Ireland, Georgia, Germany, Greece, and the UK with patients who consulted for CA-RTI in primary care.

**Method:**

Interviews were audio recorded, transcribed, and analysed using reflexive thematic analysis.

**Results:**

Interviews with 56 participants revealed that in the process of a GP making a diagnosis and treatment decision, patients valued several components, such as a physical examination, their GP enquiring about and listening to concerns, and a POCT. Yet, the visibility and relative importance of each of these components varied in the four main ways in which patients perceived the value of POCTs, including 1) test as objective evidence compared to ‘subjective‘ clinical judgment; 2) test as providing more precision; 3) test as inferior to clinical judgment; 4) test as one of the tools in the GP’s toolbox.

**Conclusion:**

The wide variation in patient perceptions about POCT for CA-RTI underscores the importance of recognising patient preferences regarding the diagnostic process. This understanding is important to ensure that POCT results optimally influence treatment decision-making, patient satisfaction, and acceptance of their care plan.

## How this fits in

While point-of-care tests (POCTs) are often seen as clinical decision tools, we need to consider their broader impact within the consultation. However, there has been limited exploration of patient perspectives on the value of these tests, and this study aims to fill this gap. Interviews with 56 participants revealed that in the process of a GP making a diagnosis and treatment decision, visibility and relative importance of POCT components varied in the four main ways in which patients perceived the value of POCTs, including: 1) test as objective evidence compared to ‘subjective‘ clinical judgment; 2) test as providing more precision; 3) test as inferior to clinical judgment; 4) test as one of the tools in the GP’s toolbox. The wide variation in patient perceptions about POCT for community-acquired acute respiratory tract infections (CA-RTI) underscores the importance of recognising patient preferences regarding the diagnostic process.

## Introduction

General practice is facing unprecedented changes. Growing threats from respiratory and/or pandemic infections and antimicrobial resistance (AMR), patient demand and concerns,^1,2^ and shortages and burnout in the GP workforce^2,3^ are some of the few challenges facing the ‘front door’ of health care. In the context of these demands, some of the core characteristics of general practice may be under threat. Specifically, patients being able to receive person-centred care and benefit from a relationship with their GP through continuity of care — defined as core characteristics of general practice — can be difficult to achieve when demand is high, time is limited, and the workforce is shrinking.

The implementation of technology and diagnostics into primary care may introduce an additional layer of complexity to the preservation of these core values and characteristics of primary care that influence care quality. Traditionally, primary care has had limited access to advanced diagnostic technology, both within local medical facilities and in terms of referrals to specialised services;^4^ however, over the last decade this landscape has been changing,^5^ and this change is expected to accelerate.

One such example is POCTs. These are tests that are performed during a consultation with a healthcare professional who has appropriate training^6^ and provide rapid ‘on site’ results that are actionable at the point of care.^7,8^ In the area of respiratory infections and antibiotic prescribing, these tests have the potential to help tailor management and advice, and to safely reduce unnecessary prescriptions, promoting self-care for self-limiting infections. They can thus contribute to tackling antibiotic resistance. In light of technological advancements, it is crucial to consider how these POCTs are implemented and consider any unintended consequences from both clinician as well as patient perspectives. Yet, previous studies have mainly focused on clinicians’ views of POCTs,^9–13^ with a slowly growing body of research exploring patient views.^14–20^ These studies showed mixed findings in relation to patient satisfaction with POCTs; some found that patients were happy to have POCTs,^16,17,19^ if their clinician felt that a POCT would be necessary,^18^ and felt that a POCT could benefit them by providing clinicians with more certainty about their diagnosis,^16,18^ and subsequently best treatment.^18,19^ POCTs were considered to be convenient if accessed outside of GP hours in pharmacies,^14,20^ or if the alternative was having to visit their GP practice again.^18,19^ In contrast, some studies highlighted patients’ concerns about invasiveness of tests,^18^ and worries about POCTs leading to delays due to time constraints in a GP consultation,^16,18^ as well as the precision of POCTs.^17^

While POCTs are often seen as clinical decision tools, we need to consider their broader impact within the consultation. This underscores the importance of aligning new diagnostics with the unique ethos of primary care, ensuring that the introduction of diagnostics complements rather than compromises its core principles. Yet, the exploration of patient views on how they perceive the value of the tests has been limited and this study addresses this important gap.

## Method

### Design and setting

This was a qualitative study, conducted as part of a process evaluation, involving semi-structured interviews with patients who participated in the intervention arm of the PRUDENCE trial. The PRUDENCE trial is a platform randomised controlled trial of point-of-care diagnostics for patients >1 year consulting primary care practices for CA-RTI in ten European countries (Belgium, France, Georgia, Germany, Greece, Hungary, Republic of Ireland, Poland, Spain, and the UK). The trial is evaluating the effectiveness of a POCT strategy to safely reduce antibiotic prescribing in primary care. Patients were randomised based on the influenza season and predominant symptom of cough or sore throat to either routine care or to a testing strategy that could involve testing for C-Reactive protein (CRP, using AFINION) test, Group A streptococcus (GAS, using BD Veritor), influenza (using BD Veritor), SARS-CoV-2 (using BD Veritor). The trial results will be reported elsewhere.

The PRUDENCE process evaluation consists of four components: interviews with patients; interviews with GPs; surveys with patients; and surveys with GPs. The interview study with patients aimed to explore patient views of POCTs for CA-RTIs and how they impact consultations. In this article, we answer the research question ‘How do patients value POCTs when consulting for CA-RTIs?’

### Participants, sampling, and recruitment

While the quantitative surveys were conducted in all countries, the qualitative interviews were only conducted in six countries (the UK, Republic of Ireland, Belgium, Germany, Greece, and Georgia). These countries were purposively selected to get variation in health system organisation and geographical location in Europe, and based on whether there was a researcher available to conduct interviews. Each country has a network coordinator responsible for managing the primary care sites taking part in the trial. We recruited patients who experienced POCTs from these sites. We used convenience and purposeful sampling to recruit patients with variation in age, symptom presentation, and type of test(s) they had received. We aimed to recruit 8–15 participants per country.

### Data collection

Seven trained qualitative primary care researchers completed interviews. All interviewers followed a topic guide with questions focusing on patients’ views of their consultation and the test(s) they received (Supplementary Box 1). Interviews took place by telephone or video, via Microsoft Teams. All participants gave verbal or written consent to take part in the study. Interviews were audio recorded and transcribed verbatim. Interviews were conducted in patients’ own language and then translated into English, where relevant, to be analysed.

### Data analysis

Data collection and analysis took place concurrently. The interviews were analysed using reflexive thematic analysis^21^ with the aim of understanding patient views of POCTs for CA-RTIs and how they impact consultations. Within this analysis, we then focused on data related to the research question on the value of POCTs. All transcripts were coded line by line by MW to create categories. These were then discussed by the core team (MW, MEH, SA and STC) and refined. These categories were then grouped to create themes speaking to the experiential aspects of patients receiving the tests. The categories and themes were created using an iterative and consensus-based approach, identifying similarities and differences among patients until data saturation was reached.

At each stage of this process, data were discussed within the core team on a monthly basis. To further ensure rigour, the ongoing analysis was discussed within the multidisciplinary study team, including all interviewers in each country, to establish an understanding of local contexts, where relevant to interpreting findings. NVivo 12 was used to facilitate data analysis. This article adheres to the Consolidated Criteria for Reporting Qualitative Research (COREQ) reporting guideline.

## Results

Fifty-six interviews were conducted between February 2022 and July 2023 and lasted between 10–36 minutes (mean 23 minutes). Basic characteristics of participants are summarised in Table 1.

**Table 1.** Key characteristics of all participants

Country	Patients, *n*	Age range, years	Female, *n* (%)	CRP test, *n* (%)	Flu test,^a^ *n* (%)	GAS test,^a^ [Table-fn T1_FN1] *n* (%)	Antibiotic prescribed, *n* (%)
UK	10	22–73	8 (80)	6 (60)	3 (30)	2 (20)	8 (80)
Republic of Ireland	12	19–79	5 (42)	5 (42)	3 (25)	7 (58)	7 (58)
Belgium	7	32–76	5 (71)	3 (43)	1 (14)	3 (43)	3 (43)
Greece	9	20–78	7 (77)	3 (77)	4 (44)	5 (55)	4 (44)
Georgia	9	21–66	9 (100)	4 (44)	5 (55)	4 (44)	3 (33)
Germany	9	24–59	7 (77)	3 (33)	6 (66)	4 (44)	0 (0)
Total	56	19–79	41 (73)	24 (43)	22 (39)	25(44)	25 (44)

a Some patients had both FLU and STREP A test.

We present four themes on patient views on the value of POCTs.

### Perceived value of POCT in a GP consultation

**Figure 1.**
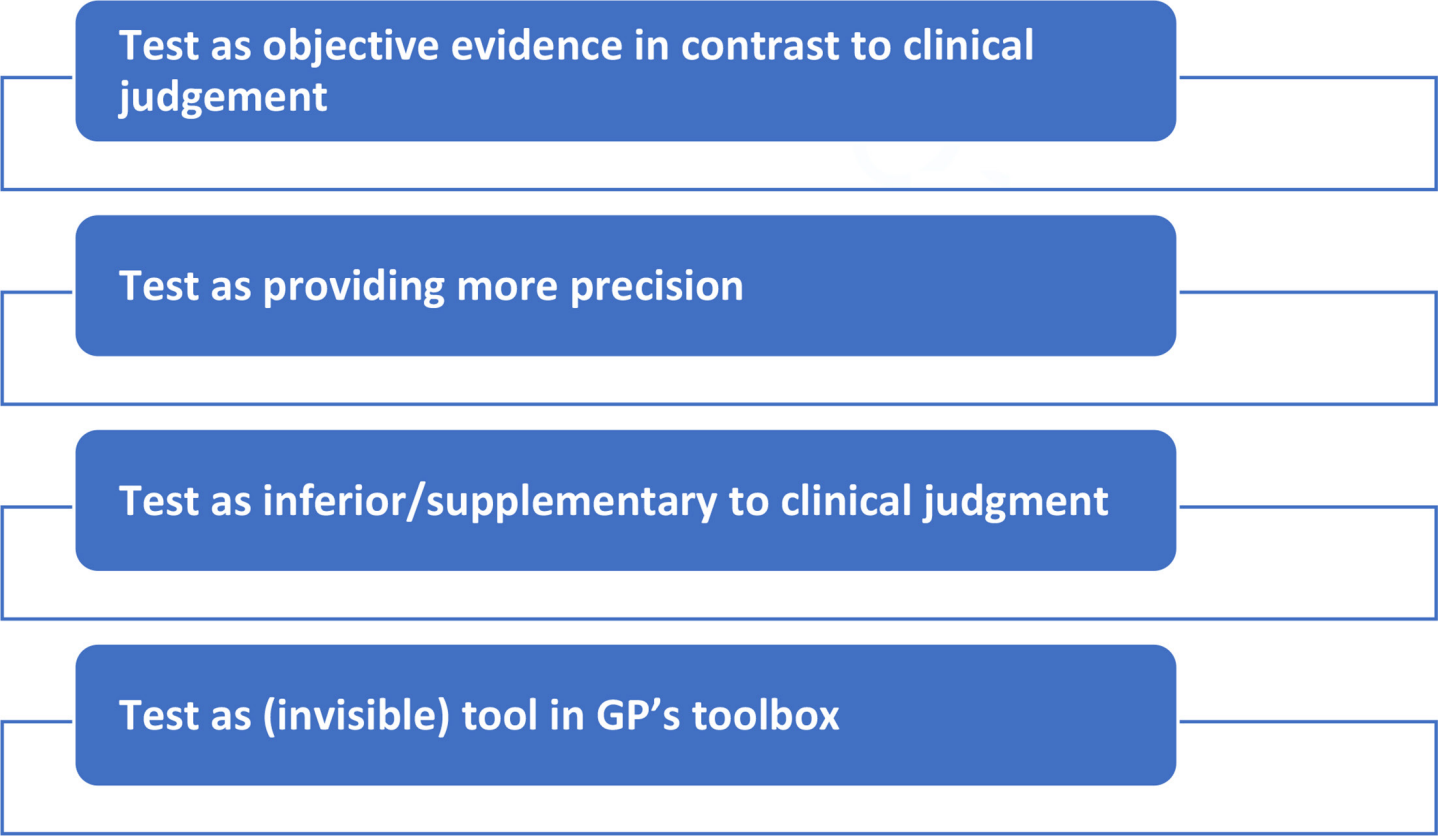
Overview of the four themes

In the process of a GP making a diagnosis, patients placed value on several components including: 1) undergoing a physical examination; 2) having their concerns listened to and addressed by their GP, and 3) undergoing a test. Yet, the visibility and relative importance of each of these components varied across the participants, highlighting diverse and interacting ways a diagnosis could be obtained. We identified four ways in which patients perceived the value of a POCT in the context of their consultation, as summarised in Figure 1, and described further below.

### Test as objective evidence in contrast to clinical judgment

Some patients placed great value on the POCTs. These patients viewed the GP as a person who has the authority to make a decision about their diagnosis and, importantly, their treatment. However, they also perceived this power as potentially misplaced, as they perceived the process of making a diagnosis by a GP as subjective. This perceived subjectivity was defined by patients as making a decision that was not based on evidence but a mere ‘opinion’, and an opinion which could easily differ from another GP based on the same clinical presentation. In contrast, a POCT was discussed as providing absolute certainty, differing from the subjective judgment of GPs regardless of the type of POCT. Thus, POCTs provided a basis to challenge an erroneous clinical judgment:

*‘Taking on a decision, that it’s not just personal. So, it is objective. So, everyone can decide the same thing instead of thinking, oh, by my point of view it’s a viral infection. Oh no, by my point of view it’s a bacterial infection. So, the outcome of a personal experience compared to a test, it’s different.* […] *So, luckily, I had the CRP test.’ (A102, 38y [years]; CRP of 29; abx [antibiotics], UK)*

While this perception seemed to be especially strong in the UK in patients who felt that they needed antibiotics and GPs felt the opposite, it was also visible in patients who did not receive an antibiotic and was also shared in the Republic of Ireland, Belgium, Ireland and to a lesser extent in Greece. These patients seemed to recognise that GPs often need to make difficult decisions and the POCT was seen as something that could help a GP, as the alternative was indeed (still) seen as ‘guessing’:

*‘Because when you don’t have to do a test, the doctor does the examination, he sees what is going on* […] *and gives antibiotics. Whereas there you get the tests and you don’t go ‘blind’, the very truth is.’ [C110, 35y; Strep A – neg, no abx, Greece]*

### Test as providing more precision

For some patients, the perceived aim of a POCT was not to replace the ‘subjective’ clinical judgment but to enhance and refine the precision of a GP’s assessment. Thus, while they placed high value on the comprehensive dialogue and thorough examination by a GP, the inclusion of any type of diagnostic test in the consultation was seen to add a layer of accuracy to the diagnostic process. This approach seemed to foster a sense of confidence and satisfaction in participants across all countries, except Georgia, as they appreciated the apparent synergy between clinical expertise and rapid diagnostic techniques. Patients in these countries therefore felt they were receiving a more comprehensive and precise healthcare experience:

*‘I felt quite positive about having something tested rather than just having an exchange of information. It strikes me as a more credible and reliable route of enquiry.’ [E108, 61y, neg [negative] flu, no abx, Ireland]*

Across all countries, this precision was often defined as knowing what GPs were treating, and in the UK, Belgium, Germany, Republic of Ireland, and Greece, was also related to whether an underlying issue needed a medication including an antibiotic, and if so, what type:

*‘I think the doctors were more knowledgeable about what I had and they knew exactly what I needed and how to deal with it. I mean, what they had to deal with.’ [C111, 59y; pos [positive] flu; no abx, Greece]*

In addition, the perception related to precision of POCT seemed to differ depending on the type of test; patients who received CRP test highlighted the precision of POCT as providing more transparency, security, and progress to the process of diagnosis whereas patients who received STREP A or flu tests tended to speak about precision by ruling in or out the illnesses they were being tested for.

### Test as inferior and/or supplementary to clinical judgment

In contrast, for a minority of patients the credibility of the consultation lay mainly in the interaction with a GP and receiving a physical examination, along with reassurance that their symptoms did not indicate a serious illness. The reassurance provided by a GP seemed to have a genuine therapeutic value, empowering patients and making them feel better able to cope with their situation. A thorough examination and reassurance (before a test was performed) was seen as sufficient to allay patients’ concerns and, consequently, as superior to the reassurance provided by a test. While a test result was perceived as valuable and could reinforce this reassurance, for these patients, the starting point was the trust put in the physical examination and the attentive care received from a GP:

*‘The GP saw me quickly and after listening to my chest he said it was very clear that I didn’t have a chest infection, which made me feel much better right away, and that he thought I just had a really nasty viral infection in my upper respiratory tract, so all in my throat essentially.* […] *I started feeling much better just having been examined in the office with him explaining to me why I was feeling the symptoms I was feeling.’ [A104, 39y; CRP of 7, no abx, UK]*

A GP was regarded as the most credible source of an accurate diagnosis for some patients; a reliance on a test result was perceived as potentially detrimental to them. Here, the power dynamic between a test and a GP shifted, emphasising the perceived necessity for the GP to adopt a holistic view and prioritise other sources of information, such as patients’ symptoms, as necessary:

*‘It is important that the machine* […] *it’s another tool in the decision making, and that it was reassuring to hear him tell me, "I think you need antibiotics regardless of what this* [test] *says, regardless of the study, I’m going to prescribe you antibiotics." So, I thought that was reassuring because it wasn’t then the machine taking over the decision making but being part of it. It validated his decision rather than determining it. That’s what you want in a doctor, you don’t want a doctor to just be determined by the stuff they have to hand, you want them to be able to make decisions based on you and what you’re presenting.’ (A107, 39y; CRP of 15; abx, UK]*

### Test as a (invisible) tool in a GP’s toolbox

Finally, for some patients the test was not seen as central to their consultation. These patients highlighted that they came to get a diagnosis and help from their GP. They described their consultation as consisting of multiple components including being physically examined, having their questions answered, being provided with reassurance, appropriate treatment, and having a test. However, all the components seemed to hold ‘equal’ value to patients:

*‘I really liked that the doctor was very attentive and gave me a lot of time. It is very important for me that the doctor gives me an adequate amount of time to understand my conditions well and not rush the procedures. I also liked the quality and speed of tests they conducted; I did not have to wait for a long time. And the fact that they came up with a good treatment plan in a short span of time was also very pleasing for me.’ [F105, 34y, neg STREP A, neg flu, no abx, Georgia*)

For some patients, especially in Greece and Georgia, the test became almost an invisible and insignificant part of the consultation. Here, patients perceived the GP as being in charge of the consultation and felt that it was up to the GP to decide what tools would be best to use. Thus, some patients seemed to only recall the test after a direct prompt and did not seem to perceive this consultation to be in any way different to previous ones. For them, the most valuable components were having their questions answered, being examined, and feeling that someone was taking them seriously, with a test often mentioned in passing:

*‘But since the doctor wanted to do it, I did it. I liked that she wanted to pay more attention to me even when I told her that I was fine. And they took a lot of care of me in the doctor’s office that day. We sat down and talked, she looked at me carefully and they measured my blood pressure, the nurses examined me too. I didn’t mind if she hadn’t done the test, but I liked that she wanted to pay attention to me.’ [C108, 66y, neg flu, no abx, Greece]*.

## Discussion

### Summary

The findings from interviews with 56 people consulting with CA- RTI in primary care in six European countries identified the multifaceted nature of patients’ perspectives on the diagnostic and treatment decision-making process. Patients valued several aspects of their consultation with a GP that were important to them, such as physical examination, clinicians enquiring about and listening to their concerns, as well as having a POCT. Yet, the visibility and relative importance of each of these components varied widely among the participants highlighting diverse ways through which patient-centred care could be delivered. We found that patients perceived POCTs in diverse ways, ranging from viewing them as objective evidence that surpasses subjective clinical judgment; considering them as tools in providing increased precision; as being potentially inferior to the nuanced expertise of clinical judgment; or seeing them as valuable but not essential additions to a GP’s toolbox.

Some patients in the UK, the Republic of Ireland, Belgium, and, to a lesser extent, Greece, viewed POCTs as objective evidence, in contrast to ‘subjective’ clinical judgment. Patients in these countries also strongly valued GPs assessments, while also recognising the precision that POCTs offer in making a diagnosis and guiding treatment decisions. Moreover, in the UK, Belgium, Germany, Republic of Ireland, and Greece, this extended to considerations about whether an underlying issue required medication, including antibiotics, and if so, which class. Of note is that patients’ views on the precision of POCTs differed depending on the test type, with CRP tests viewed as transparent and adding reassurance to clinical diagnosis, and STREP A or flu tests viewed as confirming or ruling out specific illnesses. However, a minority of patients in the UK perceived POCTs as inferior to clinical judgment. Conversely, patients primarily in Greece and Georgia regarded POCTs as tools for GPs to use at their discretion, sometimes not even mentioning them, possibly influenced by the availability of other tests less commonly performed in other countries, such as white blood cell counting.

### Strengths and limitations

This study offers unique insights on the perceived value of introducing POCT in the general practice in the context of CA-RTI. This large, multi-country, and therefore multi-health system dataset provides valuable insights into the diverse ways patients see the POCTs alongside other components of the consultations. The study benefited from interviews being conducted in patients’ own language, thus allowing the collection of rich data. They also shed light on some contextual information about general practice in different countries. Despite the overall substantial number of interviews conducted, the number of interviews in each country was relatively limited, potentially hindering the achievement of data saturation at the individual country level. Given the qualitative research design of our study, our aim was to explore, understand, and interpret patient experiences, and identify factors that shape these perspectives; thus cross-country comparisons in this perspective would benefit from further quantitative research that can measure and assess cross-cultural differences. Furthermore, recruitment was confined to practices involved in the PRUDENCE trial, which may not fully represent the typical practices in each country or capture the full spectrum of patient experiences. Data collection was conducted by researchers with varying roles within the trial, different professional backgrounds, and diverse levels of experience in conducting interviews, which could have influenced both the richness of data collected and the power dynamics during the interviews.^22^ Reflexivity was therefore crucial in this context, as it allowed us to critically reflect on our own positionality, assumptions, and interactions that may have shaped the data collection process and interpretations. The analysis was led by core team based in the UK and Belgium, potentially introducing a Cultural West-centric lens, but extensive discussions with teams in each country were undertaken to mitigate this and enhance understanding of the wider issues facing primary care across all participating countries.

### Comparison with existing literature

Previous studies focusing on patients’ views of POCTs showed that patients valued POCTs as they felt reassured about their condition^20^ and had more confidence in a diagnosis and treatment plan,^10,18^ but some highlighted the need to interpret POCT cautiously.^18^ Our study extends this by shedding light on more diverse ways in which patients saw the value of the POCTs. Firstly, our study extends understanding of the view that POCTs can provide more confidence in a diagnosis and treatment by highlighting that it is the ‘precision’ of the process that mattered to patients which was related to knowing what exactly to treat, and what treatment to receive, while also potentially reducing AMR. Secondly, it highlighted that a patient may greatly trust POCT and see it ‘in contrast’ to subjective clinical judgment of a GP. This was previously reflected in a GP-focused study where GPs felt that patients thought their clinical assessment was just ‘guesswork’.^22^ Thirdly, conversely, some patients may view POCTs as in fact inferior to clinical judgment, highlighting that trust in a test in isolation can be problematic. This has been noted before by patients describing the need to interpret the test results in conjunction with other results.^18^ Fourthly, it highlighted that POCT could be seen as a tool for the GP, to be used at their discretion, as also highlighted by others.^17^ It is important to highlight that the views around the value of the test have not seemed to largely differ depending on the result of that test, with both patients who had received an antibiotic and those who had not contributing to the four different ways in which POCT were valued.

Although POCTs were viewed differently, our results show the importance of both physical examination and acknowledging patients concerns, with open and clear communication between patients and GPs seen as valuable for all patients. This is in line with other studies showing that patients value a thorough physical examination,^23^ and GPs taking time to listen to their concerns or ask questions about their illness.^16,24–27^ However, this study highlights the importance of balance between communication skills and reliance on POCTs.

### Implications for practice

For many years, POCTs have been promoted as a key component to enhance personalised medicine by helping clinicians better target antibiotics to only those likely to benefit, and thus to help contain antimicrobial resistance.^28^ In the UK, the National AMR Action Plan^29^ and the British In Vitro Diagnostics Association Strategy^30^ have identified AMR and infectious diseases as critical areas where diagnostics could play a key role in providing solutions. As potentially easier to use, more rapid, and accurate POCTs are coming to market, successful roll out will need to build on a solid understanding and considered response to the perspectives of patients on the place of POCTs within a patient-centred consultation. Thus, integrating feedback from diverse patient populations and ensuring that these tests complement rather than replace essential aspects of care will be crucial for optimising their effectiveness and acceptance. Patients who seem to put their trust primarily in the test highlight the patient’s potential desire for concrete, measurable data to support the diagnostic process, thereby underscoring the need for transparency in the decision-making process not involving POCTs. Conversely, patients who view POCTs as inferior to the expertise and intuition of a GP’s clinical judgment show the need to reassure patients that a test is not the only way in which a diagnosis and treatment plan is made. This may facilitate the acceptance of POCTs while recognising the symbiotic relationship between clinical expertise and diagnostic tools, something which perhaps should be promoted. Others also highlighted, albeit from the GP perspective, the dangers of overusing the tests, or giving it too much weight in determining the treatment.^31,32^

While POCTs are undoubtedly valuable as clinical decision tools, their broader impact on patient-centred care, trust-building, and overall consultation experience should not be overlooked. This is especially crucial, as regardless of the extent of the role that patients ascribed to the POCTs, they all value GPs who take the time to listen to their concerns, perform thorough physical examinations, and ask relevant questions about their illness. It seems that for some patients who have less trust in their GP, the POCTs can play a greater role and therefore it is important that we invest time and efforts in rebuilding the trust if necessary. This is especially important as the study highlights that for many patients POCTs cannot and should not replace other key components of a consultation. It thus emphasises the importance of dedicating sufficient time to each patient encounter to ensure a holistic understanding of their health needs. While this study focused on the experiences of patients who consulted with GPs, the findings are likely relevant to a broader range of healthcare professionals within primary care, given the increasingly diverse workforce. Relational continuity — the ongoing, trusting relationship between patients and healthcare providers^33,34^ — may apply not only to GPs but also to nurses, pharmacists, paramedics, and other healthcare professionals involved in patient care. These relationships can bring similar benefits, including enhanced trust, improved communication, and a deeper understanding of patient needs across the different roles but can influence a consultation involving POCT in a different way. As healthcare teams become more multidisciplinary, recognising the importance of relational continuity across all provider–patient interactions is likely to become key to maintaining consistent, high quality care and patient satisfaction, regardless of which professional is involved in the consultation.

Our cross-country examination also illuminated the importance of context, particularly in relation to the healthcare systems and ways of consulting. For example, different lengths of consultations and availability of other diagnostics together with patients’ preferences, their views of general practice in general, and the extent of continuity of care may influence how they perceive the consultation and POCTs specifically.

Our findings underscore the complexity of patient perspectives on diagnostic processes using POCTs, emphasising the need for GPs to navigate these diverse viewpoints. Striking a balance between physical examination, addressing patient concerns, and POCT use is crucial. This will be vital to guarantee that POCT results play a meaningful role in GP consultations.

## Data

The dataset relied on in this article is available from the corresponding author on reasonable request
